# Eyelash Rejuvenation Using Bimatoprost 0.03%: A Prospective Pilot Split-Face Study in Indian Population

**DOI:** 10.1055/s-0045-1809150

**Published:** 2025-05-19

**Authors:** Manoj Kumar Johar, Digjeet Kaur, Pradeep Singh, Ankur Bhatia, Nishant Kumar

**Affiliations:** 1 Department of Aesthetic, Reconstructive and Plastic Surgery, Max Super Speciality Hospital, Delhi, India

**Keywords:** eyelash rejuvenation, prostaglandin, Trulush, bimatoprost

## Abstract

**Background:**

Ophthalmic bimatoprost has been used worldwide for treatment of glaucoma, and enhanced eyelash growth has been reported with such use. Dermal application of prevalent bimatoprost preparations for eyelash enhancement has been reported with adverse effects.

**Objective:**

This article assesses if revised formulation (Trulush) of bimatoprost 0.03% provides effective as well as safe eyelash enhancement in Indian population.

**Materials and Methods:**

Prospective split-face study was conducted for 12 weeks in willing subjects for their eyelash rejuvenation. Subjects completed the pre- and post-study questionnaires for subjective evaluation. Bimatoprost (Trulush) was applied on the upper eyelid margin once a day. Adverse events were also assessed. Objective evaluation was done using available digital scale and pre- and post-study photographs at 12 weeks.

**Results:**

All Subjects reported improvement in length, darkness, and density. Objective assessment corresponded with the subjective assessments. No significant adverse effects were reported by the subjects.

**Conclusion:**

Bimatoprost 0.03%, in the available formulation (Trulush), is found to be effective for topical application and eyelash rejuvenation by their increase in length, darkness, and density. There are no significant side effects.

## Introduction

Eyelash serves an important biological function and is an important aesthetic feature. The state of eyelash can affect an individual's self-image. To achieve longer, fuller eyelashes, available modalities include use of cosmetics, eyelash extensions, permanent pigment tattooing, and eyelash transplants. 1 However, there is no standardized evidence-based treatment for pharmaceutical eyelash rejuvenation.

Bimatoprost ophthalmic solution 0.03% is an ocular hypotensive agent used worldwide as a second-line treatment of open-angle glaucoma and ocular hypertension. 2 Eyelash growth was the most frequently reported adverse outcome during these studies. 3

Animal studies and recent Food and Drug Administration phase 3 clinical trials have shown that bimatoprost increases the percentage of eyelash and eyebrow follicles in the anagen phase. This results in increase in its length. It also increases the size of the dermal papilla and hair bulb resulting in thicker and fuller eyelashes. In addition, bimatoprost induced melanogenesis of eyelash follicles resulting in darker eyelashes. 4

Previous clinical trials have proven that bimatoprost 0.03% is associated with increased growth of eyelashes when applied topically over the upper eyelash line resulting in longer, thicker, and darker eyelashes. Therefore, the use of bimatoprost 0.03% has been considered for improvement in eyelash rejuvenation.

Also, previous studies focused on topical bimatoprost 0.03% to determine prominence of eyelash growth. 5 Most studies report objective assessment and do not report on the satisfaction score of subjects. 6 We did not find any studies with subjective as well as objective assessment with topical application of bimatoprost 0.03% for eyelash enhancement, especially among Indian population.

Therefore, we aim to study the efficacy and safety of topical application of bimatoprost 0.03% to the upper eyelash line for aesthetic eyelash rejuvenation among Indian population that is based upon subjective and objective evaluation and assessment.

## Materials and Methods

### Study Design

This is a prospective pilot study conducted over 12 weeks. Subjects above 15 years of age, desirous of improving their natural eyelashes were included in the study. Those excluded from the study were individuals previously diagnosed with glaucoma or ocular hypertension, any known hypersensitivity to bimatoprost 0.03%, history of any uncontrolled systemic disease or abnormality, pregnant or lactating women, or those planning a pregnancy, or not using reliable form of birth control, history of nonsurgical eyelash enhancement procedure, any previous trauma to the upper eyelid, or history of permanent makeup procedure of eyelids.

### Treatment Protocol

Informed consent was taken from all the subjects. The subjects were provided with two vials labeled “A” and “B” at the beginning of this study. Vial “A” contained only the vehicle without bimatoprost 0.03% and vial “B” contained bimatoprost 0.03% as the active agent.

Subjects were instructed to apply contents of vial “A” on the base of the right eyelash line and those of vial “B” on the base of the left eyelash line with the applicator included with the vials, in the evening, once daily for 4 weeks. At the end of 4th week the subjects were instructed to apply contents of vial “A” on the base of the left eyelash line and those of vial “B” on the base of the right eyelash line as before, in the evening, once daily for 4 weeks. They were then asked to discontinue the application at the end of 8 weeks and were called for final review at 12 weeks.

### Clinical Examination

All the subjects were reviewed pretreatment (baseline), at 4 weeks, 8 weeks, and then at 12 weeks (end of study). At each review the subjects reported on apparent improvement and adverse effects if any. Digital images were also taken at each of these review points under standard conditions with DSLR (digital single-lens reflex) camera. All the above data was transferred onto a master sheet.

### Photographic Documentation and Analysis

The digital images of the subjects were documented and used for comparison of all three parameters of length, darkness, and density using ImageJ software. The scores obtained were averaged for each follow-up. The mean average for the length, darkness, and density of eyelashes was calculated. Mean average for eyelash satisfaction as per the questionnaire for pretreatment and at 12 weeks was calculated.

### Health Outcome Questionnaire

To track adverse effects health outcome questionnaire was provided to the patients. Subjects were asked to answer the questionnaire at weeks 4, 8, and 12.

### Eyelash Satisfaction Questionnaire

To assess subjects' satisfaction with the treatment and subjective evaluation of their eyelashes appearance at 12th week, the Eyelash Satisfaction Questionnaire (ESQ) was given to the patients at each follow-up.

## Results

### Patient Demographics

Nine out of 10 patients enrolled in the study were women. Based on the medical histories collected all subjects were in good general health.

### Adverse Effects

Subjects 1, 4, and 6 reported burning sensation after 3rd and 2nd follow-up, respectively. As per subject 1 the stinging lasted for 1 to 2 minutes and was barely noticeable. Subjects 4 and 6 reported that the stinging lasted for less than a minute and was barely noticeable. Remaining 7 subjects did not report any stinging/burning.

### Questionnaire Responses

Subjects reported significantly greater improvements from baseline. Upon final review at 12 weeks' follow-up, 7 subjects reported overall significant improvement, 2 subjects reported marked improvement, and 1 subject reported moderate improvement in all three parameters.

**Fig. 1:**
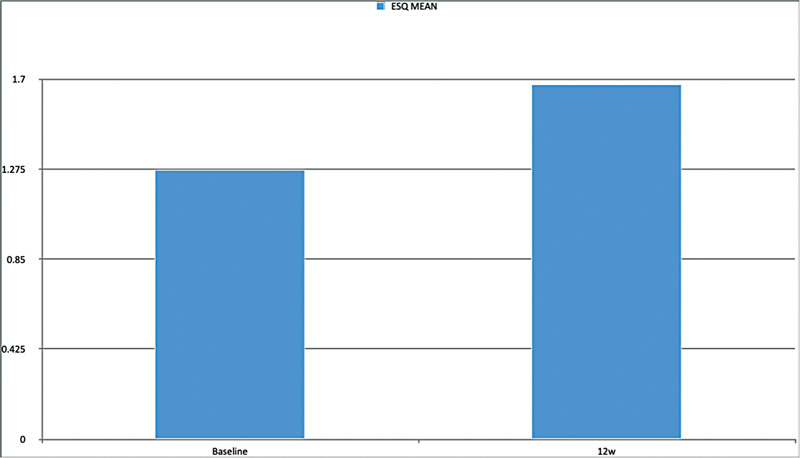
The graph depicts the mean change in the Eyelash Satisfaction Questionnaire from baseline (pretreatment) to the 12th week (posttreatment follow-up) in 10 patients. At baseline, the mean score was 1.273, while at the 12th week, it increased to 1.674. The improvement in mean change reflects subjective satisfaction in all three parameters (length, darkness, and density).

Subjective evaluation determining the patient satisfaction with respect to length, density, and darkness of the eyelashes:

Fig. 1 represents mean change in eyelash satisfaction questionnaire from baseline to 12th week.

Photographic assessment:

Objective evaluation using ImageJ analysis software determining the length, darkness, density of the eyelash.

**Fig. 2:**
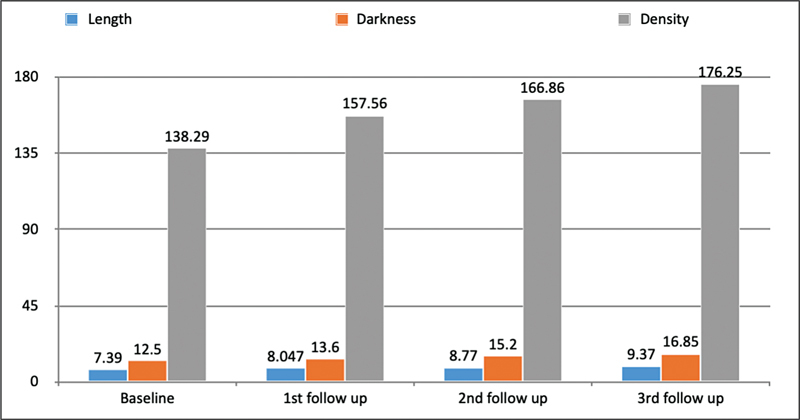
The graph represents the objective evaluation of all three parameters at baseline, 1st follow-up (4th week), 2nd follow-up (8th week), and 3rd follow-up (12th week) by comparing change in mean at different follow-ups. The average mean at baseline for eyelash length, darkness, and density was 7.39, 12.5, and 138.29, respectively, whereas after 12 weeks it was increased to 9.37, 16.85, and 176.25, respectively.

Fig. 2 : Mean change from baseline to third follow-up in eyelash length, darkness, and density.

**Fig. 3:**
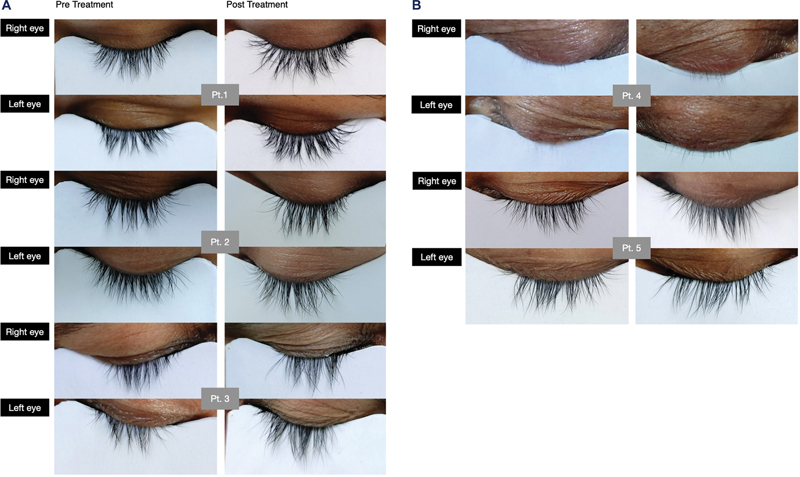
Representation of the effects of bimatoprost in patients 1, 2, and 3 ( **A** ). Representation of the effects of bimatoprost in patients 4 and 5 ( **B** ).

Fig. 3A and 3B represents effect of Bimatoprost on eyelash length, darkness and density at 12th week on 5 study subjects.

## Discussion

Bimatoprost 0.03% is an ophthalmic solution used for the treatment of glaucoma and ocular hypertension. It was accidentally found to result in eyelash enhancement as a side effect in patients of glaucoma.

A research paper by Johnstone has highlighted hypertrichosis and pigmentation effects linked to prostaglandin analogs like latanoprost, providing foundational knowledge of prostaglandin-induced follicular activity. 7 Further supporting this, a clinical trial reported that 4% of patients suffered from redness and itching in the eye after application of LATISSE (bimatoprost 0.03%). 8 However, the Wester et al study provided important safety data, finding no significant ocular adverse events in subjects who completed the study. While there were no periocular or iris pigmentation changes documented during study visits, two patients noted subjective periocular changes between visits, which they self-treated and which did not recur. Importantly, these changes were not deemed significant by the patients themselves. 9

In contrast, our study on Trulush 0.03% revealed distinct safety outcomes. None of our subjects reported redness and itching after the application of Trulush. Three out of 10 patients reported a stinging sensation after its application for 1 to 2 minutes, which subsided on its own. However, a larger subject cohort needs to be studied to validate these findings. Additionally, variations in application, duration of application, and persistence of results after discontinuation need further investigation.

While safety and tolerability form a crucial part of the evaluation, subject satisfaction is equally important in assessing product efficacy. The ESQ findings ( Fig. 1 ) demonstrate a significant improvement in subject satisfaction from baseline to the 12th week. The mean ESQ score increased from approximately 1.273 at baseline to 1.674 at 12 weeks, indicating a noticeable enhancement in perceived eyelash length, density, and overall satisfaction. These results suggest that Trulush 0.03% effectively meets user expectations for eyelash enhancement.

In terms of formulation, Trulush 0.03% offers a key point of differentiation compared with established formulations like LATISSE and Lumigan. Both LATISSE and Lumigan formulations rely on bimatoprost 0.03% as the active ingredient, supplemented with similar excipients such as benzalkonium chloride, sodium chloride, sodium phosphate, and citric acid. Trulush, while containing the same concentration of bimatoprost, differentiates itself by incorporating sodium hyaluronate, a known humectant. This addition enhances the product's hydrating properties, potentially improving user comfort and tolerability. To truly assess the efficacy and safety of Trulush 0.03% relative to established formulations, comparative studies are essential.

## Conclusion

Bimatoprost 0.03%, in the available formulation (Trulush), is found to be effective for eyelash rejuvenation by topical application and demonstrates subjective as well as objective increase in length, darkness, and density of the eyelashes. There are no significant associated side effects.
